# Upper Extremity and Musculoskeletal Injuries Related to Videogame and Augmented Reality Game Usage: A Narrative Review Article

**DOI:** 10.1007/s12178-025-09956-9

**Published:** 2025-02-13

**Authors:** Christopher M. Belyea, Gregory Rummel, Nathan Lanham, Kevin P. Krul

**Affiliations:** 1 Department of Orthopaedic Surgery and Rehabilitation, Womack Army Medical Center, 2817 Rock Merritt Avenue, Fort Liberty, NC 28310 USA; 2 Department of Surgery, Uniformed Services University of the Health Sciences, 4301 Jones Bridge Rd, Bethesda, MD 20814 USA; 3 Department of Orthopaedics and Rehabilitation, The Pennsylvania State University, 30 Hope Drive, Suite 2200, Hershey, PA 17033 USA; 4 Department of Orthopaedic Surgery, Tripler Army Medical Center, 1 Jarrett White Rd, Honolulu, HI 96859 USA

**Keywords:** Videogame injury, Motion-controlled videogame, Musculoskeletal injury

## Abstract

**Purpose of Review:**

Since the introduction of videogames and augmented reality technology, injuries associated with e sports have garnered increased attention from researchers and healthcare professionals. This review articles examines the spectrum of injuries associated with videogames and augmented reality and describes the nuances of the diagnoses associated with gaming injuries.

**Recent Findings:**

Videogames changed architecture from a sedentary style of playing to a more interactive console with the introduction of technologies such as the Wii or Pokémon Go. This new style of gaming increased the range of musculoskeletal injuries to include lower energy injuries and more severe high energy trauma because of the introduction of mobility causing distraction and a neglect for safety.

**Summary:**

The previous sedentary nature of gaming lends itself to prolonged periods of static posture contributing to long-term effects and potential health implications in the future. This review article presents current literature on the specific injury types and increasing need for awareness of videogame-related injuries to further highlight preventative measures needed to mitigate injury risk and promote healthy gaming habits and ergonomic awareness.

## Introduction

The videogame industry has evolved considerably over the last half a century. The origin of videogames began in the late 1950s when, using one of the Brookhaven National Laboratory’s large research computers, an American physicist created the first interactive computer game title, Tennis for Two [1]. This early videogame included rudimentary controllers with a turning knob and a button [1]. It wasn’t until the 1970s that the first commercial home interactive videogame consoles became available with the Magnavox Odyssey’s Brown Box and Atari’s Pong and Video Computer System 2600 [2]. Both of these early home console systems increased the complexity of the human-computer interfaces with joysticks, controllers and even a light gun [2]. Videogaming interfaces over the next several decades remained largely unchanged with the introduction of the Sega, Nintendo, and PlayStation systems. It was during this time that upper extremity overuse injuries became regularly identified [3].

The video gaming industry saw another paradigm shift in 2006 with the introduction of Nintendo Wii [4]. As a motion-controlled video gaming system, the Wii videogames are played by acting out realistic whole-body sports movements [3]. Microsoft Xbox 360 Kinect and PlayStation Move followed with similar interfaces. While motion-controlled gaming promoted physical activity, it also has been associated with a higher risk for injuries. In fact, some of these injuries have been of similar nature and severity to what is seen in the real sports that they simulate. [3] Additionally, the orthopaedic injury profiles expanded to include lower extremity ailments. The lack of stretching, strength training and warm up exercises normally practiced by real sports athletes, are all thought to contribute to the motion-controlled video gaming injuries [5].

The gaming industry saw yet another fundamental evolution to a new level of escapism with the maturation of the virtual reality headsets and the augmented reality game environments. In 2016, Pokémon Go was introduced as an augmented reality game and within 1 week of its release boasted over 65 million participants [6]. Pokémon Go is played on smart phones, whereby the virtual game environment was overlaid onto the users’ real word surroundings. In augmented reality the users manipulate their avatars to move around the game’s map, and virtual locations corresponds to real world structures and soundings linked via global positioning satellites (GPS) [7, 8]. With this new version of user gaming interface, the injury risk profiles have evolved as well. Higher energy trauma, including motor vehicle accidents, has been reported with augmented reality game usage attributed to users ambulating and even driving without regard to their surroundings [9].

## Demographics

It is estimated that 183 million people play videogames at least an hour a day in the United States [6]. Male players account for 60% of all videogame players [10]. The average age of video game players has been reported to be around 35years of age [10]. According to the Pew Research Center 97% of American teenagers aged 12 to 17 reported playing videogames [10]. It should be noted that older demographics also participate in videogame play on a regular basis: an estimated 26% of all players are age 50 and above [11].

A national database inquiry study of the NEISS database indicated that in emergency room visit related to videogames, 52% of patients were between ages 6–15 years of age, and those 30 years and older represented only 10% of patients. The majority of patients were female and account for 62% of presentations. Of the presenting injuries, 29% of were strains and sprains, and 58% of injuries involved the upper extremity. This database study suggests that those presenting to emergency rooms are younger, more likely to be female, and most likely to complain of an upper extremity injury [11].

### Upper Extremity Injuries

Upper extremity injuries account for the majority of videogame related injuries [11]. The thumbs, for its dexterity and degrees of freedom of motion, is often the primary modality by which participants interface with videogame controllers. Overuse injuries of the thumb include the so called “PlayStation thumb” or formerly the “Atari-finger” [12, 13]. This syndrome involves skin blistering, swelling, and numbness caused by friction from fast pace repetitive button pressing [13]. Other exertional injuries include a form of “Nintendinitis”, which has been described to involve either a flexor pollicis longus (FPL) tendonitis or an extensor pollicis longus (EPL) tendonitis caused by rapid prolonged manipulation of the controller’s buttons [14, 15]. These repetitive overuse injuries have been reported in adolescent and childhood patients and respond well to non-operative treatment of nonsteroidal anti-inflammatory medication (NSAIDs) and activity modification [13, 15].

It has been proposed that videogame players may be at risk for these repetitive injuries due to the pain perception dampening that immersed video-gamers experience [16]. This pain dampening videogame phenomenon has been well studied in pediatric patients during burn treatments and may be due to activation of the neuroendocrine hypothalamic-pituitary-adrenal axis [16].

Repetitive gaming motion has also been attributed to complete ruptures of the FPL and EPL tendons and rupture of the metacarpal phalangeal joint volar plate. The site of attritional rupture of the EPL tendon has been identified at the level of Lister’s tubercle and can be associated with extensor carpi radialis brevis (ECRB) partial rupture and tendonitis [17]. These acute injuries have been reported in adult patients and require operative repair and/or reconstruction of ruptured structures [16, 18].

In motion-controlled videogames, participants may become less aware of their surroundings and strike nearby household future. This mechanism has resulted in a myriad of upper extremity injuries including a severe acute fracture of the thumb [19]. Acknowledging these injuries, the videogaming industry has made protective gloves available.

Repetitive hand and wrist injuries, such as central palmar ulceration [20, 21], “joystick digit,” and “Space Invaders Thumb” can result from repetitive long-term manipulation of the joystick. “Joystick Digit,” is a long or ring finger tenosynovitis resulting in palpable flexor tendon nodules and triggering of the effected digits in otherwise young healthy patients [24]. The “Joystick Digit” has been responsive to corticosteroid tendon sheath injections [22]. “Space-Invaders Wrist”, is a nonspecific wrist joint capsular strain, extensor digitorum communis (EDC) tenosynovitis and DeQuervains stenosing tenosynovitis [23–25]. A commonly cited risk factor for hand and wrist level injuries is repetitive play sessions lasting for at least six to eight hours [21, 25].

Videogame play has also been associated with upper extremity neuropathies. Ulnar nerve neuropathy has been described as a result of prolonged gaming. “Videogame Palsy”, a distal ulnar neuropathy of the deep motor branch can result from compression at the level of Guyon’s canal. “Videogame Palsy” can be associated with hypothenar callus formation from repetitive game-controller induced trauma [26]. Cubital tunnel and carpal tunnel syndrome have also been correlated with prolonged videogaming play [27].

The widespread participation in the motion-controlled videogaming has led to a rise in shoulder and arm related pathology. “Wii Shoulder” or “Wiiitis” is upper arm and shoulder girdle muscle group pain and arm swelling resulting from prolong participation in sports simulated motion-controlled games [28–30]. Simulated tennis and bowling games have been identified as risk factors for the development of “Wii Shoulder.” [30]. It is unclear if this represents a cumulative effect of an overuse injury or a more acute delayed-onset muscle soreness. MRI evaluation has demonstrated selective muscle group edema without frank tears or intramuscular hematoma while laboratory analysis has been significant for mild increases in serum creatine kinase (> 8,000 U/L).^28,31^ It has been proposed that motion-controlled videogame usage is not limited by the participants’ strength or endurance as the associated force magnitudes are relatively low. The motion-controlled handheld controllers often weigh less than 200 g [31]. It is thought that prolonged, aggressive simulated sports movements may lead to abnormal arm deacceleration forces [31]. NSAIDs and rest has been the mainstay treatment for shoulder and upper arm videogame-related edema [28].

## Lower Extremity Injuries

With the wide spread of motion-controlled sport simulation games, particularly tennis and bowling, lower extremity injuries resulting from videogame participation have been described [31, 32]. The severity of these injuries correlate with the intensity of the athletic sport which the videogames simulate. The knee appears to be particularly prone to injury. “Wii knee” has been described as a lateral dislocation of the patella occurring with a twist and planting motion of the leg, such as seen with simulated motion-controlled tennis videogame [31, 33]. This lateral patella dislocation from videogame play has been associated with osteo-chondral fractures requiring operative repair [33]. Additional surgically treated knee injuries which have been reported include medial meniscus tears from simulated bowling and anterior cruciate ligament tear from simulated boxing [3, 34].

The ankle and foot have also been described as being vulnerable to injury during motion-controlled video gaming, although these injuries are mostly treated non-operatively. In particular, the Achilles tendon pathology named “Achilles Wiitis”, a partial Achilles tendon tear, has been described in middle-aged participants of motion-controlled videogames [35]. Complete Achilles tendon ruptures and base of the 5th metatarsal fractures have also been reported [35–37].

## Augmented Reality Injuries

Where cell phones, videogaming and global positioning system meet, a new cultural phenomena of augment reality was born. When Pokémon GO was released in 2016 it was lauded for encouraging active rather than sedentary videogame play [6]. The cardiovascular benefit and weight loss benefit has been contrasted to the risk profile associated with negotiating the real word surroundings while distracted by videogame participation [8, 38, 39]. Surveys of augmented reality game players have highlighted the potential risks, with over 40% of players endorsing play while riding bicycles and almost a third of players endorsing videogame participation while driving a vehicle [7]. Augmented reality gaming has increased the severity of gaming-related trauma. Not only have pedestrian versus motor vehicle accidents been attributed to augment reality videogame play, but high-energy traumatic injuries from motor vehicle accidents have also been reported in the literature, including traumatic brain injuries, long bone injuries, and pelvic fractures [9, 40, 41]. It is important for physicians treating traumatic injuries to recognize that augmented-reality videogames may provide a risky distraction for high energy trauma.

## Summary

It is important to understand injury risk profiles associated with videogame participation to better diagnose, treat and prevent related musculoskeletal pathology. While future research is needed, including prospective studies monitoring rates of injury amongst the e-sports professional, videogaming continues to be a safe endeavor. As the industry and user gaming interface has evolved from handheld controllers to augmented reality, so has the injury pattern and injury severity profiles.

**Box 1:** Risk Factors for Videogame Related Injuries

• 6–15 years of age	
• Female • Upper extremity complaints • Greater than 6 h of daily videogame play	

**Table 1:** Videogame related injuries

Injury		Citation
**Upper Extremity**		
Playstation thumb	*thumb flexion contracture*	Vaidya, *Lancet*, 2004
Thumb Nintendinitis	*thumb flexor tendonitis*	Cassadova, *JHS Am*, 1991
Rupture of thumb volar plate		Mandal, *Injury*, 2005
Thumb Metacarpal Injury		Galanopoulos et al., *BMJ*, 2012
Extensor Pollicis Longus Tendon Rupture		Bhangu et al., *JHS Am*, 2009
Joystick Digit	*ring and long finger tenosynovitis and triggering*	Oysterman, *JAMA*, 1987
Ulcerative Nintendinitis	*palmer ulceration from repetitive joystick rubbing*	Koh *MJA*, 2000; *Wood*, *BMJ*, 2001
Dequervains stenosing tenosynovitis		Reinstein, *APMR*, 1983
Space Invaders Wrist	*stiff painful wrist*	McCowan, *NEJM*, 1981
Nintendonitis Hand	*dorsal swelling of hand, EDC reactive tenosynovitis*	Siegel, *Orthopedics*, 1991;
Hand vibration syndrome		Cleary, *BMJ*, 2002
Nintendo Elbow	*Elbow pain*	Bright, *BMJ*, 2008
Pac Man Elbow	*Tendonitis at the flexor or extensor wad origin*	Skow, *Time*, 1982
Videogame palsy	*distal ulnar neuropathy in a video game ethensuist*	Friedland, *NEJM*, 1984
Slot Machine Tendinitis	*long head of the biceps pain*	Neiman, *NEJM*, 1981
Acute wiitis	*isolated infra-spinatus tendonitis*	Bonis, *NEJM*, 2007
Wii shoulder	*tiredness and a sore shoulder*	Cowley BMJ 2008
Wii-knees	*arm swelling and muscle weakness*	Baxter and Madhok, SMJ, 2011
**Lower Extremity**		
Wii Knee	*patella dislocation*	Robinson et al., 2008
Wii Knee	*meniscal injury from simulation 10-pin bowling*	Almedghio et al., 2009
Anterior Cruciate Ligament Rupture		Muller, *Medicine*, 2015
Achilles’ wiiitis	*Achilles’ tendonitis resulting in pain or rupture*	Beddy et al., *AJR*, 2009
**Other trauma**		
Wii Spine	*Clay shovelers fracture*	Brown and McKenna, *SWJ*, 2009
Nintendo neck	*Neck pain associated with prolonged play*	Miller, *CMAJ*, 1991
Internal Carotid artery dissection		Faivre et al., *Neurology*, 2009
Nintendo epilepsy/ Space Invaders epilepsy	*Seizure induced by videogame flashing screen*	Hart, *NEJM*, 1990; Rushton, *Lancet, 1981*
eThrombosis	*Lower extremity venous thromboembolism associated with immobility*	Beasley, *Eur Respir J*, 2003
Wii Eye Injury	*self inflicted glob rupture*	Razavi and lam, *JAAPOS*, 2011

## How this Article was Created

This article was created initially using a personal archive of references with both authors having treated many videogame injuries. A multi-modal search through our library system was conducted utilizing Medline (OVID or Pubmed) CINAHL (OVID), CAB Abstracts, Embase, FEDRIP, NIH RePORTER, BRD, DTIC, Chochrane Library TRIP Database, and Google Scholar. Articles were reviewed for applicability to this topic. Key search terms included “Videogame Injury,” “Electronic Sports Injury,” “E-Sports Injury” and using brand names of popular gaming platforms in Medline.

### How Patients were Involved in the Creation of this Article

#### N/A

##### Education into Practice

- For patients with tendinopathy or chronic injuries of the upper extremity: Do you screen for hobbies including computer usage, typing, video gaming, weightlifting and sports that may contribute to the root cause?
- Do you routinely counsel adult patients on consideration of sedentary screen time including videogame usage?
- Do you discuss the potential for photosensitivity from videogames, computer usage or television in patients with seizure disorders?
- When is the last time you asked a patient about how they spend their evenings and weekends to screen for activities that may not be normally listed under “hobbies.”

### Contributorship and the Guarantor

CMB and KPK conceived the article and are guarantors. All authors wrote and reviewed the article, created the boxes, and helped with the figures.

## Data Availability

No datasets were generated or analysed during the current study.

## Declarations

### Licence

The Corresponding Author has the right to grant on behalf of all authors and does grant on behalf of all authors, an non-exclusive licence on a worldwide basis to Current Reviews in Musculoskeletal Medicine Publishing Group Ltd to permit this article (if accepted) to be published in Current Reviews in Musculoskeletal Medicine and any other Current Reviews in Musculoskeletal Medicine product and sublicences such use and exploit all subsidiary rights, as set out in our licence (https://authors.bmj.com/policies/#copyright*).*
